# Heterocycles 38. Biocatalytic Synthesis of New Heterocyclic Mannich Bases and Derivatives

**DOI:** 10.3390/molecules200712300

**Published:** 2015-07-06

**Authors:** Denisa Leonte, László Csaba Bencze, Csaba Paizs, Florin Dan Irimie, Valentin Zaharia

**Affiliations:** 1 Department of Organic Chemistry, “Iuliu Haţieganu” University of Medicine and Pharmacy, 41 Victor Babeş street, RO-400012 Cluj-Napoca, Romania; E-Mail: hapau.denisa@umfcluj.ro; 2 Department of Biochemistry and Biochemical Engineering, Babeş-Bolyai University of Cluj-Napoca, 11 Arany János street, RO-400028 Cluj-Napoca, Romania; E-Mails: cslbencze@chem.ubbcluj.ro (L.C.B.); paizs@chem.ubbcluj.ro (C.P.); irimie@chem.ubbcluj.ro (F.D.I.)

**Keywords:** Mannich reaction, heterocyclic compounds, lipase, biocatalytic promiscuity

## Abstract

This paper describes the biocatalytic synthesis of new Mannich bases containing various heterocyclic rings (thiazole, furane, thiophene, pyridine) by applying the lipase catalyzed trimolecular condensation of the corresponding heterocyclic aldehydes with acetone and primary aromatic amines, in mild and eco-friendly reaction conditions. The obtained Mannich bases were acylated to their corresponding N-acetyl derivatives. All compounds were characterized by ^1^H-NMR, ^13^C-NMR and MS spectrometry.

## 1. Introduction

Mannich bases and derivatives are pharmacologically and chemically useful compounds due to their therapeutic potential and to the reactivity of their functional groups [1,2]. In addition, aromatic heterocyclic ring systems have a great value in the field of medicinal chemistry, being responsible for a variety of biological activities. In particular, the thiazole ring is a pharmacophore group intensely studied due to its remarkable biological potential, including anti-inflammatory [3], antitumor [4], antibacterial [5], and antiviral [6] properties. The thiophene and furane moieties have attracted special interest in the development of new potent analgesic [7], anti-inflammatory [8], antimicrobial [9], and anticonvulsant [8] agents. The pyridine ring is often found in the structure of antimicrobial agents, the most relevant example being the antitubercular agent isonicotinic hydrazide.

The classical variant of the Mannich reaction involves the use of formaldehyde, a secondary amine hydrochloride and an enolisable carbonyl compound as CH-acidic substrate. The drawbacks of the classical procedure are more evident when primary amines or ketones with two reactive alpha positions are used in the reaction. In this case, the formation of secondary products cannot be avoided, because of the lack of selectivity. Modern variants of the Mannich reaction have been proposed, either starting from pre-formed enolates [10], immonium salts [11], or by organometallic/organocatalytic methods [12]. Despite the notable successes achieved, the use of these chemical methods is generally limited by economical reasons, by the low stability of the intermediates and because of the toxicity of the solvents, intermediates and catalysts used.

Biocatalytic processes have become a useful and green alternative in organic synthesis due to their multiple advantages. Moreover, the enzymes can be immobilized and this way they can be readily separated from the reaction mixture and reused in many cycles. Previous studies reported that, besides their natural role, lipases are able to catalyze different synthetic reactions, such as C-C and C-N bond forming [13]. The lipase-catalyzed direct Mannich reaction using as substrates some substituted benzaldehydes, primary amines and ketones was previously described and a possible mechanism was also proposed [14]. The method afforded Mannich bases that cannot be obtained by classical Mannich reaction. However, at present, there is a lack of information in this field and further studies are necessary to be made, in order to extend the applicability of this biocatalytic approache.

As a continuation of research devoted to the development of mild and eco-friendly biocatalytic synthetic routes and being aware of the pharmacological value of nitrogen, sulfur and oxygen containing heterocycles [3,4,5,6,7,8,9], we decided to exploit the potential of the lipase-catalyzed direct Mannich reaction by implementing for the first time this enzymatic reaction in the series of heterocyclic compounds. The novel obtained heterocyclic Mannich bases could have promising applications in medicinal chemistry, due to the therapeutic potential of the heterocyclic side chain and his multiple functionalization.

## 2. Results and Discussion

The aim of this study was to exploit the biocatalytic promiscuity of lipases to promote the trimolecular Mannich type condensation, in order to obtain new compounds with biological potential. We oriented our research to the extension of this methodology in the series of heterocyclic compounds. The first goal was to find the optimal reaction conditions for the enzymatic condensation between acetone, primary aromatic amines and various heterocyclic aldehydes. In order to investigate the substrate scope of this method, different types of heterocyclic aldehydes were used as substrates: 2-arylthiazole-4-carbaldehyde, furane-2-carbaldehyde, thiophene-2-carbaldehyde and pyridine-3-carbaldehyde. Thiazolic aldehydes **1a**–**d** were synthesized as previously described in the literature, by Hantzsch condensation of various thiobenzamides with 1,3-dichloroacetone, followed by Sommelet reaction [15].

First, various lipases have been evaluated as biocatalysts for the direct Mannich condensation between thiazolic aldehydes, aniline and acetone, in aqueous media (Table 1). Kun Li *et al.* previously demonstrated that the presence of water in the reaction media strongly influence the activity of lipases in the Mannich type condensation. In their experiments, the optimal water concentration was found to be between 40% and 50% [14]. Based on these reported data and taking into consideration the low solubility of heterocyclic aldehydes in water, we choose an acetone/water 1:1 (*v*/*v*) system as the reaction media, in order to increase the solubility of substrates.

**Table 1:** Lipase screening for the three-component Mannich type condensation of 2-phenylthiazole-4-carbaldehyde with aniline and acetone. Reaction time: 20 h. 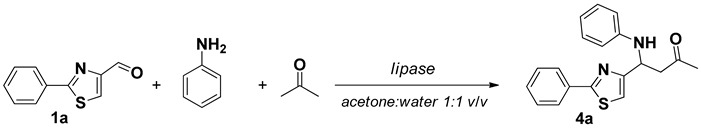

Entry	Enzyme	Yield (%)
1	No enzyme	0
2	Lipase from *Burkholderia cepacia*	8
3	Lipase from *Candida rugosa*	72
4	Lipase from *Pseudomonas fluorescens* (AK free)	62
5	Lipase B from *Candida antarctica* (CaL-B)	82
6	Lipase A from *Candida antarctica* (CaL-A)	7
7	Bovine serum albumin	0

Lipases from *Candida rugosa*, *Pseudomonas fluorescens* and lipase B from *Candida antarctica* exhibited good catalytic activity in the experiments, the products being obtained in 62%–82% yields after 20 h (Table 1, entries 3–5). In order to confirm the catalytic activities of lipases in the Mannich reaction, control experiments were also performed. In the absence of the enzyme or in the presence of bovine serum albumin, the formation of Mannich bases was not detected. Lipase B from *Candida antarctica* (CaL-B, Novozym 435) was chosen for further studies.

Encouraged by these results, we decided to investigate also the ability of lipases to accept thiazole-derived methylketones as CH-acidic substrates, instead of acetone. Consequently, the condensation of benzaldehyde with aniline and 4-methyl-2-phenylthiazol-5-yl ethanone was investigated, but in this case the lipases were not active.

**Scheme 1:**
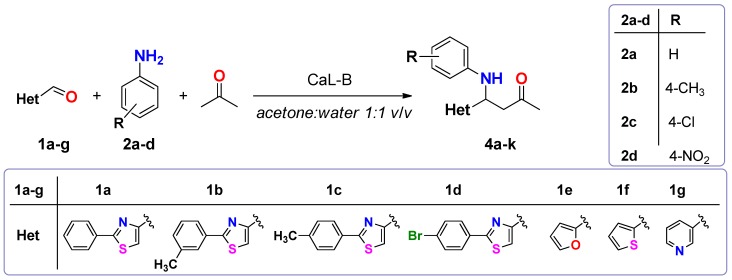
CaL-B catalyzed Mannich type condensation between heterocyclic aldehydes, primary aromatic amines and acetone.

The ability of CaL-B to transform other heterocyclic aldehydes in the Mannich type condensation was also investigated. Furan-2-carbaldehyde, thiophene-2-carbaldehyde, pyridine-3-carabaldehyde and various substituted 2-phenylthiazole-4-carbaldehydes were accepted as substrates in the CaL-B catalyzed trimolecular condensation with aniline and acetone (Scheme 1).

Further experiments were oriented on the condensation of heterocyclic aldehydes with acetone and other primary aromatic amines (*p*-toluidine, *p*-Cl-aniline, *p*-nitroaniline), using CaL-B as biocatalyst. Similar good results were obtained when *p*-toluidine was used as substrate. CaL-B was inactive in the enzymatic Mannich reactions involving *p*-Cl-aniline and *p*-nitroaniline (Table 2, entries 9 and 10).

**Scheme 2:**
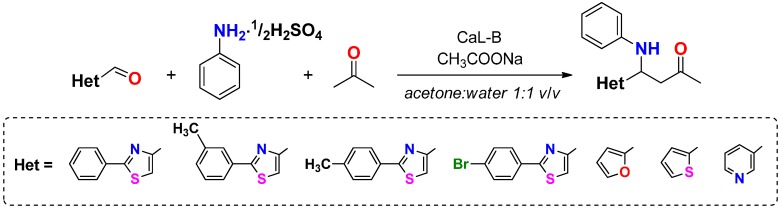
CaL-B catalyzed direct Mannich reaction, using heterocyclic aldehydes, acetone and aniline sulfate (1.1 eq.) as substrate.

Because amines are generally susceptible to oxidative degradation and their purification is sometimes laborious, we opted for the use of the corresponding amine salt, because of its better stability and its easier manipulation and storage. Similar good results were obtained when aniline sulfate was used in the enzymatic reactions, in the presence of an equimolar quantity of sodium acetate (Scheme 2).

The preparative scale enzymatic synthesis of Mannich bases **4a**–**k** was performed, in the presence of CaL-B as biocatalyst. The enzymatic reactions were conducted at room temperature, under continuous shaking at 1200 rpm. The aromatic amine was added in a small excess (1.1 eq.), thus ensuring the total conversion of the heterocyclic aldehyde. The Mannich bases **4a**–**k** were obtained in 66%–80% yield, starting from the corresponding aldehydes (0.075 mol/L), aromatic amine (0.0825 mol/L), in the presence of CaL-B (Novozym 435), in an acetone/water 1/1 (*v*/*v*) mixture (Table 2). Compound **4a** was easily isolated, because it precipitated in the reaction media, and no purification was necessary. The other enzymatic reaction products **4b**–**k** were isolated by extraction and purified on column chromatography. By measuring the optical rotations of the obtained Mannich bases, it was found that the enzymatic reactions occurred without enantioselectivity.

**Table 2:** Preparative scale enzymatic synthesis of thiazolic Mannich bases **4a**–**k** ^1^. 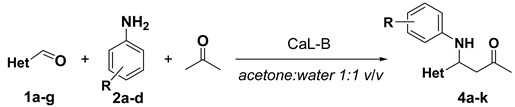

Entry	Het	R	Substrate		Product	Reaction time	Yield (%) ^2^
1	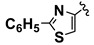	H	**1a**	**2a**	**4a**	48 h	75
2	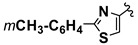	H	**1b**	**2a**	**4b**	48 h	70
3	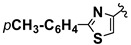	H	**1c**	**2a**	**4c**	48 h	68
4	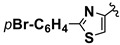	H	**1d**	**2a**	**4d**	72 h	66
5	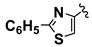	4-CH_3_	**1a**	**2b**	**4e**	48 h	72
6	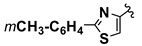	4-CH_3_	**1b**	**2b**	**4f**	48 h	68
7	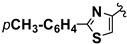	4-CH_3_	**1c**	**2b**	**4g**	48 h	69
8	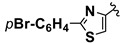	4-CH_3_	**1d**	**2b**	**4h**	72 h	66
9	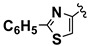	4-Cl	**1a**	**2c**	**-**	48 h	-
10	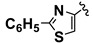	4-NO_2_	**1a**	**2d**	**-**	48 h	-
11	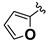	H	**1e**	**2a**	**4i**	48 h	74
12	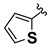	H	**1f**	**2a**	**4j**	48 h	76
13	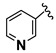	H	**1g**	**2a**	**4k**	48 h	80

^1^ A solution of aldehyde **1a**–**g** (0.075 mol/L) and aromatic primary amine (0.0825 mol/L) dissolved in an acetone/water 1/1 *v*/*v* mixture (20 mL) was shaken at 1200 rpm in the presence of 150 mg CaL-B at room temperature. ^2^ Isolated yields were calculated considering theoretical 100% conversion of the aldehyde **1a**–**g**.

**Scheme 3:**
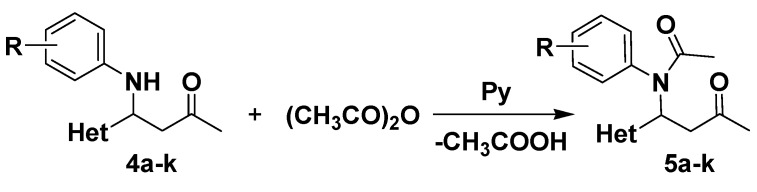
Synthesis of *N*-acetylated Mannich bases **5a**–**k**.

In order to increase the stability of the obtained Mannich bases, further acylation of these compounds was performed, using acetic anhydride and a catalytic amount of pyridine, affording compounds **5a**–**k** in good yields (Scheme 3).

All synthesised compounds were purified, physically and chemically characterized by ^1^H-NMR, ^13^C-NMR and MS spectroscopy.

In the ^1^H-NMR spectra of Mannich bases **4a**–**k**, all signals corresponding to the protons located on the benzene rings are present in the aromatic region. The proton located in the 5th position of the thiazole ring appears as a singlet at 7.08–7.15 ppm. The C*H* proton located in the β position of the butanone chain appears as a triplet at 4.89–5.18 ppm, while the C*H*_2_ protons from the α position are appearing as a doublet, at chemical shifts of 2.96–3.17 ppm. In the ^1^H-NMR spectra of the corresponding *N*-acylated derivatives **5a**–**k**, the β C*H* proton gives a triplet at 6.41–6.47 ppm, while the α C*H*_2_ protons are appearing as a doublet of doublet of doublets, at chemical shifts between 3.05 and 3.11 ppm.

In the ^13^C-NMR spectra of compounds **4a**–**k**, a characteristic signal between 206 and 208 ppm indicates the presence of the carbonyl group. In the case of *N*-acetylated compounds **5a**–**k**, two characteristic carbonyl signals are present, indicating the presence of the ketone group (205–207 ppm for *C*=O) and, respectively, the presence of the amide group (170–170.9 ppm for *C*O-NH). All aromatic and aliphatic signals are also present, thus confirming the chemical structures of the products.

The MS spectra of the Mannich bases and their *N*-acylated derivatives are revealing similar fragmentation pathways. The chemical structures are confirmed by the presence of the molecular peak, which in most cases is accompanied by the peak [M + H]^+^.

## 3. Experimental Section

### 3.1. Analytical Methods

The ^1^H-NMR and ^13^C-NMR spectra were recorded on a Brucker Avance DPX-300 spectrometer operating at 600 and 150 MHz, respectively. Chemical shifts on the δ scale are expressed in ppm values from TMS as internal standard. GC-MS measurements were performed with a Shimadzu QP 2010 Plus machine (Shimadzu Europa GmbH, Duisburg, Germany). EI-MS spectra were recorded with the spectrometer operating at 70 eV.

Thin layer chromatography (TLC) was carried out using Merck Kieselgel 60F_254_ sheets (Merck, Darmstadt, Germany). Spots were visualized in UV light. Preparative chromatographic separations were performed using column chromatography on Merck Kieselgel 60Å (63–200 μm).

Melting points were determined on open glass capillaries using an Electrothermal IA 9000 digital apparatus (Bibby Scientific Limited, Staffordshire, UK).

### 3.2. Reagents and Solvents

The commercial chemicals and solvents were products of Sigma Aldrich or Fluka (Sigma Aldrich Chemie GmbH, Steinheim, Germany; Fluka production GmbH, Buchs, Switzerland). All solvents were purified and dried by standard methods as required. Lipase B from *Candida antarctica* (CaL-B, Novozym 435) was purchased from Novozymes (Bagsvaerd, Denmark). Lipase from *Candida rugosa* (CrL) was purchased from Fluka. Lipases from *Pseudomonas fluorescens* and *Burkholderia cepacia* (BcL) were from Amano, England.

### 3.3. Enzymatic Synthesis of Heterocyclic Mannich Bases

#### 3.3.1. Lipase Screening for the Enzymatic Mannich Reaction

To a solution of 2-phenylthiazol-4-carbaldehyde **1a** (14.16 mg, 75 μmol) dissolved in acetone (600 μL), aniline (7.5 μL, 82.5 μmol), deionized water (600 μL) and different lipases (15 mg) were added in this order. The reaction mixtures were shaken at 500 rpm for 20 h and then analyzed by TLC using a mixture of petroleum ether: ethyl acetate 4:1 (*v*/*v*) as eluent. The enzyme was removed by centrifugation and filtration and then it was washed three times with acetone in order to recuperate the product. The filtrate was brought to dryness by rotatory evaporation under reduced pressure. The crude product was purified by column chromatography, using a mixture of petroleum ether: ethyl acetate 4:1 (*v*/*v*). The purified product was dried and weighed.

#### 3.3.2. Analytical Scale CaL-B Catalyzed Direct Mannich Reaction Using Different Aldehydes and Aromatic Amines as Substrates

To a solution of 2-arylthiazol-4-carbaldehyde **1a**–**d** (25 μmol) dissolved in acetone (200 μL), aromatic primary amine (27.5 μmol of aniline sulfate/*p*-toluidine/4-chloroaniline/4-nitroaniline), deionized water (200 μL) and CaL-B (4 mg) were added in this order. In the case when aniline sulfate was used as substrate, sodium acetate (2.26 mg, 27.5 μmol) was added to the reaction mixture. The reaction mixtures were shaken at 500 rpm for 20 h and then analyzed by TLC using a mixture of petroleum ether: ethyl acetate 4:1 (*v*/*v*) as eluent.

#### 3.3.3. Preparative Scale Enzymatic Synthesis of Thiazolic Mannich Bases **4a**–**k**

To a solution of heterocyclic aldehyde **1a**–**g** (1.5 mmol of 2-arylthiazole-4-carbaldehyde, furane-2-carbaldehyde, thiophene-2-carbaldehyde, pyridine-3-carbaldehyde) dissolved in acetone (10 mL), aromatic primary amine (1.65 mmol of aniline sulphate/p-toluidine), deionized water (10 mL) and CaL-B (150 mg) were added in this order. When aniline sulphate was used as substrate, sodium acetate (136 mg, 1.65 mmol) was added to the reaction mixture. The reaction mixtures were shaken at 1200 rpm until complete consumption of aldehydes **1a**–**g** (2–3 days, checked by TLC using a mixture of petroleum: ethyl acetate 4:1 (*v*/*v*) as eluent). After completion of the reaction, the enzyme was removed by filtration and washed three times with acetone in order to recuperate the product. The filtrate was concentrated under reduced pressure, in order to remove the acetone. The aqueous phase was extracted three times with dichloromethane. The organic phase was dried over anhydrous Na_2_SO_4_ and the solvent was evaporated under reduced pressure. The crude product was purified by column chromatography, using as eluent a mixture of petroleum ether: ethyl acetate 4:1 (*v*/*v*) for compounds **4a**–**h**, dichloromethane for compounds **4i**,**j** and respectively a mixture of dichloromethane:acetone 2:1 (*v*/*v*) for compound **4k**. The obtained Mannich bases were stored in sealed flasks, at 0 °C.

*4-(Phenylamino)-4-(2-phenylthiazol-4-yl)butan-2-one* (**4a**) white solid, yield: 75%, mp 117 °C. ^1^H-NMR (300 MHz, CDCl_3_) δ 7.95–7.92 (m, 2H), 7.47–7.43 (m, 3H), 7.20–7.13 (m, 3H), 6.76–6.69 (m, 3H), 5.13 (t, *J* = 5.8 Hz, 1H), 4.62 (s, 1H), 3.17 (d, *J* = 6.1 Hz, 2H), 2.16 (s, 3H). ^13^C-NMR (75 MHz, CDCl_3_) δ 207.76, 168.52, 158.10, 146.72, 133.72, 130.17, 129.44, 129.07, 126.57, 118.28, 114.96, 113.98, 51.20, 48.35, 31.02. GC-MS: *m*/*z* found: 322 (M calculated for C_19_H_18_N_2_OS: 322); *m*/*z* (%) = 323 (M + 1, 3), 322 (M, 13), 279 (18), 266 (20), 265 (100), 188 (26), 162 (10), 121 (10), 104 (15), 93 (8), 77 (33), 65 (13), 43 (90), 39 (6).

*4-(Phenylamino)-4-(2-m-tolylthiazol-4-yl)butan-2-one* (**4b**) oily liquid, yield: 70%, ^1^H-NMR (600 MHz, CDCl_3_) δ 7.77 (s, 1H), 7.72 (d, *J* = 7.8 Hz, 1H), 7.33 (t, *J* = 7.6 Hz, 1H), 7.24 (d, *J* = 7.6 Hz, 1H), 7.18 (t, *J* = 7.9 Hz, 2H), 7.11 (s, 1H), 6.74 (t, *J* = 7.3 Hz, 1H), 6.69 (d, *J* = 7.8 Hz, 2H), 5.14 (t, *J* = 6.1 Hz, 1H), 3.17 (d, *J* = 6.2 Hz, 2H), 2.43 (s, 3H), 2.16 (s, 3H). ^13^C-NMR (151 MHz, CDCl_3_) δ 207.68, 168.72, 157.96, 146.69, 138.76, 133.56, 130.93, 129.36, 128.91, 127.04, 123.75, 118.19, 114.77, 113.93, 51.17, 48.33, 30.90, 21.43. GC-MS: *m*/*z* found: 336 (M calculated for C_20_H_20_N_2_OS: 336); *m*/*z* (%) = 336 (M, 3), 293 (3), 279 (15), 243 (40), 228 (89), 214 (26), 200 (47), 186 (13), 160 (9), 149 (100), 135 (16), 116 (10), 93 (43), 83 (17), 77 (23), 65 (24), 43 (70), 39 (58), 27 (9).

*4-(Phenylamino)-4-(2-p-tolylthiazol-4-yl)butan-2-one* (**4c**) oily liquid, yield: 68%, ^1^H-NMR (400 MHz, CDCl_3_) δ 7.82 (d, *J* = 8.1 Hz, 2H), 7.24 (d, *J* = 8.5 Hz, 2H), 7.19–7.15 (m, 2H), 7.08 (s, 1H), 6.73 (t, *J* = 7.3 Hz, 1H), 6.69 (d, *J* = 7.7 Hz, 2H), 5.13 (t, *J* = 6.1 Hz, 1H), 3.16 (d, *J* = 6.1 Hz, 2H), 2.39 (s, 3H), 2.15 (s, 3H). ^13^C-NMR (101 MHz, CDCl_3_) δ 207.67, 168.62, 157.85, 146.70, 134.82, 131.05, 129.66, 129.37, 126.43, 118.18, 114.36, 113.94, 51.18, 48.32, 30.92, 21.48. GC-MS: *m*/*z* found: 336 (M calculated for C_20_H_20_N_2_OS: 336); *m*/*z* (%) = 336 (M, 7), 293 (8), 279 (42), 243 (48), 228 (93), 215 (6), 200 (70), 135 (13), 116 (16), 93 (44), 83 (20), 77 (17), 65 (24), 43 (100), 39 (66), 27 (3).

*4-(2-(4-Bromophenyl)thiazol-4-yl)-4-(phenylamino)butan-2-one* (**4d**) semisolid, yield: 66%, ^1^H-NMR (600 MHz, CDCl_3_) δ 7.79 (d, *J* = 8.5 Hz, 2H), 7.56 (d, *J* = 8.5 Hz, 2H), 7.18 (t, *J* = 7.9 Hz, 2H), 7.14 (s, 1H), 6.74 (t, *J* = 7.3 Hz, 1H), 6.69 (d, *J* = 7.8 Hz, 2H), 5.14 (t, *J* = 6.1 Hz, 1H), 3.16 (d, *J* = 6.2 Hz, 2H), 2.16 (s, 3H). ^13^C-NMR (151 MHz, CDCl_3_) δ 207.43, 167.03, 158.34, 146.59, 132.52, 132.10, 129.37, 127.87, 124.26, 118.25, 115.23, 113.89, 51.02, 48.24, 30.84. GC-MS: *m*/*z* found: 400 (M calculated for C_19_H_17_BrN_2_OS: 400); *m*/*z* (%) = 402 (M, 1, ^81^Br), 400 (M, 1, ^79^Br), 359 (1, ^81^Br), 357 (1, ^79^Br), 345 (2, ^81^Br), 343 (2, ^79^Br), 309 (18, ^81^Br), 307 (18, ^79^Br), 294 (35, ^81^Br), 292 (34, ^79^Br), 266 (13, ^81^Br), 264 (11, ^79^Br), 149 (20), 93 (17), 83 (18), 43 (100), 39 (27), 27 (6).

*4-(p-Tolylamino)-4-(2-phenylthiazol-4-yl)butan-2-one* (**4e**) oily liquid, yield: 72%, ^1^H-NMR (600 MHz, CDCl_3_) δ 7.96–7.94 (m, 2H), 7.46–7.43 (m, 3H), 7.13 (s, 1H), 7.00 (d, *J* = 8.2 Hz, 2H), 6.63 (d, *J* = 8.4 Hz, 2H), 5.12 (t, *J* = 6.1 Hz, 1H), 3.16 (d, *J* = 6.2 Hz, 2H), 2.25 (s, 3H), 2.16 (s, 3H). ^13^C-NMR (151 MHz, CDCl_3_) δ 207.66, 168.34, 158.26, 144.37, 134.72, 133.67, 130.67, 130.06, 129.86, 128.97, 126.48, 115.29, 51.52, 48.35, 30.86, 20.46. GC-MS: *m*/*z* found: 336 (M calculated for C_20_H_20_N_2_OS: 336); *m*/*z* (%) = 336 (M, 6), 293 (4), 279 (29), 243 (3), 229 (46), 214 (100), 201 (7), 186 (39), 160 (13), 106 (21), 91 (11), 83 (37), 77 (35), 65 (11), 43 (24), 39 (73), 27 (5).

*4-(p-Tolylamino)-4-(2-m-tolylthiazol-4-yl)butan-2-one* (**4f**) oily liquid, yield: 68%, ^1^H-NMR (600 MHz, CDCl_3_) δ 7.76 (s, 1H), 7.72 (d, *J* = 7.7 Hz, 1H), 7.32 (t, *J* = 7.6 Hz, 1H), 7.24 (d, *J* = 7.6 Hz, 1H), 7.11 (s, 1H), 6.98 (d, *J* = 8.2 Hz, 2H), 6.62 (d, *J* = 8.4 Hz, 2H), 5.10 (t, *J* = 6.2 Hz, 1H), 3.15 (d, *J* = 6.2 Hz, 2H), 2.43 (s, 3H), 2.23 (s, 3H), 2.16 (s, 3H). ^13^C-NMR (151 MHz, CDCl_3_) δ 207.73, 168.66, 158.18, 144.39, 138.76, 134.79, 133.61, 130.90, 129.86, 128.90, 127.05, 123.76, 114.73, 114.23, 51.59, 48.42, 30.88, 21.44, 20.47. GC-MS: *m*/*z* found: 350 (M calculated for C_21_H_22_N_2_OS: 350); *m*/*z* (%) = 350 (M, 6), 293 (23), 243 (57), 228 (100), 215 (7), 200 (46), 135 (7), 91 (18), 83 (25), 65 (12), 43 (33), 39 (20).

*4-(p-Tolylamino)-4-(2-p-tolylthiazol-4-yl)butan-2-one* (**4g**) oily liquid, yield: 69%, ^1^H-NMR (600 MHz, CDCl_3_) δ 7.84 (d, *J* = 8.0 Hz, 2H), 7.25 (d, *J* = 8.0 Hz, 2H), 7.09 (s, 1H), 7.00 (d, *J* = 8.2 Hz, 2H), 6.64 (d, *J* = 8.3 Hz, 2H), 5.12 (t, *J* = 6.2 Hz, 1H), 3.16 (d, *J* = 6.2 Hz, 2H), 2.41 (s, 3H), 2.25 (s, 3H), 2.16 (s, 3H). ^13^C-NMR (151 MHz, CDCl_3_) δ 207.65, 168.49, 157.99, 144.32, 140.21, 130.99, 129.78, 129.58, 127.35, 126.36, 114.28, 114.16, 51.49, 48.31, 30.77, 21.39, 20.40. GC-MS: *m*/*z* found: 350 (M calculated for C_21_H_22_N_2_OS: 350); *m*/*z* (%) = 350 (M, 7), 293 (25), 243 (56), 228 (100), 215 (6), 200 (44), 135 (6), 91 (15), 83 (24), 65 (14), 43 (35), 39 (18).

*4-(p-Tolylamino)-4-(2-(4-bromophenyl)thiazol-4-yl)butan-2-one* (**4h**) white solid, yield: 66%, mp: 123–124 °C. ^1^H-NMR (400 MHz, CDCl_3_) δ 7.80 (d, *J* = 8.5 Hz, 2H), 7.56 (d, *J* = 8.5 Hz, 2H), 7.15 (s, 1H), 7.00 (d, *J* = 8.2 Hz, 2H), 6.63 (d, *J* = 8.2 Hz, 2H), 5.11 (t, *J* = 6.1 Hz, 1H), 3.16 (d, *J* = 6.1 Hz, 2H), 2.24 (s, 3H), 2.16 (s, 3H). ^13^C-NMR (101 MHz, CDCl_3_) δ 207.43, 166.94, 158.49, 144.20, 132.55, 132.08, 129.85, 127.86, 127.57, 124.22, 115.21, 114.22, 51.46, 48.28, 30.80, 20.45. GC-MS: *m*/*z* found: 414 (M calculated for C_20_H_19_BrN_2_OS: 414); *m*/*z* (%) = 416 (M, 2, ^81^Br), 414 (M, 2, ^79^Br), 359 (10, ^81^Br), 357 (10, ^79^Br), 309 (4, ^81^Br), 307 (4, ^79^Br), 294 (9, ^81^Br), 292 (9, ^79^Br), 268 (6, ^81^Br), 266 (8, ^79^Br), 191 (13), 149 (52), 106 (100), 84 (8), 77 (14), 43 (35), 27 (7).

*4-(Furan-2-yl)-4-(phenylamino)butan-2-one* (**4i**) yellow solid, mp: 69 °C, yield: 74%. ^1^H-NMR (600 MHz, CDCl_3_) δ 7.34 (s, 1H), 7.18 (t, *J* = 7.9 Hz, 2H), 6.75 (t, *J* = 7.3 Hz, 1H), 6.68 (d, *J* = 8.4 Hz, 2H), 6.29 (dd, *J* = 3.1, 1.9 Hz, 1H), 6.19 (d, *J* = 3.2 Hz, 1H), 5.04 (t, *J* = 6.3 Hz, 1H), 3.05 (dd, *J* = 16.4, 6.4 Hz, 1H), 3.00 (dd, *J* = 16.4, 6.4 Hz, 1H), 2.14 (s, 3H). ^13^C-NMR (151 MHz, CDCl_3_) δ 206.78, 154.74, 146.53, 141.70, 129.31, 118.42, 113.96, 110.42, 106.41, 48.32, 47.23, 30.67. GC-MS: *m*/*z* found: 229 (M calculated for C_14_H_15_NO_2_: 229); *m*/*z* (%) = 229 (M, 1); 143 (1); 134 (2); 92 (2); 77 (3); 65 (6); 43 (100); 39 (12); 27 (7).

*4-(Phenylamino)-4-(thiophen-2-yl)butan-2-one* (**4j**) yellow solid, mp: 70 °C, yield: 76%. ^1^H-NMR (600 MHz, CDCl_3_) δ 7.21–7.10 (m, 3H), 6.96 (d, *J* = 3.5 Hz, 1H), 6.92 (dd, *J* = 5.0, 3.5 Hz, 1H), 6.73 (t, *J* = 7.3 Hz, 1H), 6.65 (d, *J* = 7.7 Hz, 2H), 5.18 (t, *J* = 6.2 Hz, 1H), 3.08 (dd, *J* = 16.4, 6.3 Hz, 1H), 3.02 (dd, *J* = 16.4, 6.3 Hz, 1H), 2.15 (s, 3H). ^13^C-NMR (151 MHz, CDCl_3_) δ 206.75, 147.32, 146.56, 129.39, 127.04, 124.32, 124.09, 118.62, 114.16, 50.90, 50.48, 31.03. GC-MS: *m*/*z* found: 245 (M calculated for C_14_H_15_NOS: 245); *m*/*z* (%) = 245 (M, 1); 188 (2); 139 (6); 118 (4); 92 (7); 77 (2); 43 (100); 39 (6); 27 (4).

*4-(Phenylamino)-4-(pyridin-3-yl)butan-2-one* (**4k**) white solid, mp: 125 °C, yield: 80%. ^1^H-NMR (600 MHz, CDCl_3_) δ 8.64 (d, *J* = 2.0 Hz, 1H), 8.48 (dd, *J* = 4.7, 1.4 Hz, 1H), 7.70 (dd, *J* = 6.3, 1.6 Hz, 1H), 7.22 (dd, *J* = 7.8, 4.8 Hz, 1H), 7.09 (t, *J* = 7.9 Hz, 2H), 6.68 (t, *J* = 7.3 Hz, 1H), 6.53 (d, *J* = 7.8 Hz, 2H), 4.89 (t, *J* = 6.3 Hz, 1H), 2.96 (dd, *J* = 6.3, 1.9 Hz, 2H), 2.12 (s, 3H). ^13^C-NMR (151 MHz, CDCl_3_) δ 206.44, 148.78, 148.47, 146.35, 138.13, 134.34, 129.33, 123.76, 118.37, 113.88, 52.01, 50.66, 30.75. GC-MS: *m*/*z* found: 240 (M calculated for C_15_H_16_N_2_O: 240); *m*/*z* (%) = 240 (M, 22); 206 (13); 191 (53); 183 (100); 167 (7); 149 (28); 146 (50); 132 (69); 123 (14); 118 (10); 106 (23); 104 (58); 93 (21); 77 (38); 43 (55); 39 (15); 27 (7).

### 3.4. Synthesis of N-Acetylated Mannich Bases **5a**–**k**

To a solution of Mannich base **4a**–**k** (0.6 mmol) in anhydrous dichloromethane (4 mL), acetic anhydride (3 mmol) and 1 drop of pyridine were added. The reaction mixture was stirred overnight. After completion of the reaction (checked by TLC using dichloromethane as eluent), the reaction mixture was quenched with water. The organic layer was dried over anhydrous Na_2_SO_4_ and the solvent was evaporated under reduced pressure. The crude product was purified by column chromatography on silicagel, in a gradient separation procedure, using a mixture of dichloromethane: acetone from 25:1 to 9:1 (*v*/*v*) for compounds **5a**–**j** and respectively a mixture of dichloromethane:acetone 9:2 *v*/*v* for compound **5k**.

*N-Acetyl-4-(phenylamino)-4-(2-phenylthiazol-4-yl)butan-2-one* (**5a**): yellow liquid, yield: 81%, ^1^H-NMR (600 MHz, CDCl_3_) δ 7.90–7.77 (m, 2H), 7.46–7.36 (m, 3H), 7.35–7.28 (m, 3H), 7.22 (s, 1H), 7.15–6.88 (m, 2H), 6.46 (t, *J* = 7.5 Hz, 1H), 3.10 (ddd, *J* = 24.6, 16.6, 7.5 Hz, 2H), 2.13 (s, 3H), 1.79 (s, 3H). ^13^C-NMR (151 MHz, CDCl_3_) δ 205.89, 170.59, 167.23, 155.74, 140.24, 133.67, 130.05, 129.94, 129.29, 128.97, 128.43, 126.52, 116.90, 51.62, 45.81, 30.28, 23.60. GC-MS: *m*/*z* found: 364 (M calculated for C_21_H_20_N_2_O_2_S: 364); *m*/*z* (%) = 365 (M + 1, 2.7), 364 (M, 8.2), 321 (82.2), 279 (100), 265 (30.0), 188 (33.7), 93 (28.3), 77 (10.4), 43 (23.9), 39 (8.4).

*N-Acetyl-4-(phenylamino)-4-(2-m-tolylthiazol-4-yl)butan-2-one* (**5b**): yellow liquid, yield: 81%, ^1^H-NMR (600 MHz, CDCl_3_) δ 7.66 (s, 1H), 7.51 (d, *J* = 7.9 Hz, 1H), 7.36–7.27 (m, 4H), 7.22 (d, *J* = 7.5 Hz, 1H), 7.19 (s, 1H), 7.13–6.98 (m, 2H), 6.47 (t, *J* = 7.4 Hz, 1H), 3.10 (ddd, *J* = 24.6, 16.5, 7.5 Hz, 2H), 2.40 (s, 3H), 2.14 (s, 3H), 1.80 (s, 3H). ^13^C-NMR (151 MHz, CDCl_3_) δ 206.02, 170.69, 167.56, 155.65, 138.78, 133.65, 130.92, 130.01, 129.33, 128.92, 128.47, 127.19, 123.78, 119.96, 116.83, 51.74, 45.89, 30.30, 23.64, 21.51. GC-MS: *m*/*z* found: 378 (M calculated for C_22_H_22_N_2_O_2_S: 378); *m*/*z* (%) = 379 (M + 1, 0.2), 378 (M, 1), 335 (14), 293 (24), 279 (6), 202 (11), 149 (2), 135 (5), 118 (8), 93 (12), 77 (10), 43 (100), 39 (10).

*N-Acetyl-4-(phenylamino)-4-(2-p-tolylthiazol-4-yl)butan-2-one* (**5c**): yellow liquid, yield: 80%, ^1^H-NMR (600 MHz, CDCl_3_) δ 7.74 (d, *J* = 8.1 Hz, 2H), 7.32 (s, 3H), 7.21 (d, *J* = 8.0 Hz, 2H), 7.17 (s, 1H), 7.13–6.94 (m, *J* = 23.7, 16.5, 11.5 Hz, 2H), 6.45 (t, *J* = 7.5 Hz, 1H), 3.10 (ddd, *J* = 24.6, 16.5, 7.5 Hz, 2H), 2.38 (s, 3H), 2.14 (s, 3H), 1.80 (s, 3H). ^13^C-NMR (151 MHz, CDCl_3_) δ 206.00, 170.62, 167.47, 155.54, 140.36, 131.08, 129.99, 129.67, 129.30, 128.43, 126.50, 119.91, 116.42, 51.73, 45.87, 30.31, 23.64, 21.54. GC-MS: *m*/*z* found: 378 (M calculated for C_22_H_22_N_2_O_2_S: 378); *m*/*z* (%) = 378 (M, 1), 335 (6), 293 (13), 279 (4), 202 (13), 149 (22), 135 (4), 118 (3), 93 (11), 77 (5), 43 (100), 39 (16).

*N-Acetyl-4-(2-(4-bromophenyl)thiazol-4-yl)-4-(phenylamino)butan-2-one* (**5d**): white solid, mp: 127 °C, yield: 78%, ^1^H-NMR (600 MHz, CDCl_3_) δ 7.71 (d, *J* = 8.4 Hz, 2H), 7.54 (d, *J* = 8.5 Hz, 2H), 7.39–7.29 (m, 3H), 7.25 (s, 1H), 7.16–6.88 (m, 2H), 6.43 (t, *J* = 7.4 Hz, 1H), 3.11 (ddd, *J* = 24.5, 16.7, 7.5 Hz, 2H), 2.14 (s, 3H), 1.80 (s, 3H). ^13^C-NMR (151 MHz, CDCl_3_) δ 205.89, 170.73, 166.04, 156.04, 140.32, 132.22, 129.96, 129.38, 128.54, 128.01, 124.37, 122.41, 117.32, 51.71, 45.88, 30.33, 23.64. GC-MS: *m*/*z* found: 442 (M calculated for C_21_H_19_BrN_2_O_2_S: 442); *m*/*z* (%) = 444 (M, 1, ^81^Br), 442 (M, 1, ^79^Br), 401 (5, ^81^Br), 399 (5, ^79^Br), 359 (6, ^81^Br), 357 (6, ^79^Br), 345 (2, ^81^Br), 343 (2, ^79^Br), 310 (1, ^81^Br), 308 (1, ^79^Br), 149 (4), 93 (18), 77 (15), 43 (100), 39 (12), 27 (2).

*N-Acetyl-4-(p-tolylamino)-4-(2-phenylthiazol-4-yl)butan-2-one* (**5e**): yellow liquid, yield: 76%, ^1^H-NMR (600 MHz, CDCl_3_) δ 7.94–7.79 (m, *J* = 6.5, 2.7 Hz, 2H), 7.46–7.35 (m, *J* = 4.9 Hz, 3H), 7.21 (s, 1H), 7.16–7.05 (m, 2H), 7.02–6.75 (m, 2H), 6.45 (t, *J* = 7.5 Hz, 1H), 3.08 (ddd, *J* = 24.6, 16.6, 7.5 Hz, 2H), 2.33 (s, 3H), 2.14 (s, 3H), 1.79 (s, 3H). ^13^C-NMR (151 MHz, CDCl_3_) δ 205.97, 170.81, 167.17, 155.86, 138.35, 137.50, 133.71, 130.04, 129.92, 129.63, 128.96, 126.54, 116.88, 51.47, 45.81, 30.30, 23.55, 21.19. GC-MS: *m*/*z* found: 378 (M calculated for C_22_H_22_N_2_O_2_S: 378); *m*/*z* (%) = 379 (M + 1, 3), 378 (M, 2), 335 (9), 293 (10), 279 (4), 230 (4), 188 (10), 149 (4), 91 (4), 77 (7), 65 (5), 43 (100), 39 (8).

*N-Acetyl-4-(p-tolylamino)-4-(2-m-tolylthiazol-4-yl)butan-2-one* (**5f**): yellow liquid, yield: 75%, ^1^H-NMR (600 MHz, CDCl_3_) δ 7.65 (s, 1H), 7.38 (d, *J* = 8.3 Hz, 1H), 7.29 (t, *J* = 7.6 Hz, 1H), 7.22 (d, *J* = 7.5 Hz, 1H), 7.18 (s, 1H), 7.11–7.08 (m, *J* = 8.2 Hz, 2H), 7.01–6.71 (m, *J* = 46.4 Hz, 2H), 6.46 (t, *J* = 7.5 Hz, 1H), 3.09 (ddd, *J* = 24.6, 16.5, 7.5 Hz, 2H), 2.40 (s, 3H), 2.34 (s, 3H), 2.14 (s, 3H), 1.80 (s, 3H). ^13^C-NMR (151 MHz, CDCl_3_) δ 206.07, 170.90, 167.51, 155.67, 138.75, 133.88, 130.91, 129.93, 129.67, 129.53, 128.90, 127.19, 123.80, 120.11, 116.79, 51.55, 45.86, 30.28, 23.55, 21.49, 21.20. GC-MS: *m*/*z* found: 392 (M calculated for C_23_H_24_N_2_O_2_S: 392); *m*/*z* (%) = 393 (M + 1, 1), 392 (M, 2), 349 (14), 307 (14), 293 (6), 243 (5), 228 (13), 202 (3), 149 (21), 107 (28), 91 (9), 77 (3), 65 (9), 43 (100), 39 (22).

*N-Acetyl-4-(p-tolylamino)-4-(2-p-tolylthiazol-4-yl)butan-2-one* (**5g**): yellow liquid, yield: 76%, ^1^H-NMR (600 MHz, CDCl_3_) δ 7.73 (d, *J* = 8.0 Hz, 2H), 7.19 (d, *J* = 7.7 Hz, 2H), 7.14 (s, 1H), 7.12–6.74 (m, 4H), 6.45 (t, *J* = 7.5 Hz, 1H), 3.06 (ddd, *J* = 24.6, 16.5, 7.5 Hz, 2H), 2.36 (s, 3H), 2.32 (s, 3H), 2.13 (s, 3H), 1.78 (s, 3H). ^13^C-NMR (151 MHz, CDCl_3_) δ 205.96, 170.71, 167.29, 155.63, 140.21, 138.26, 137.46, 131.08, 129.84, 129.61, 129.58, 126.42, 116.29, 51.44, 45.77, 30.23, 23.49, 21.45, 21.13. GC-MS: *m*/*z* found: 392 (M calculated for C_23_H_24_N_2_O_2_S: 392); *m*/*z* (%) = 393 (M + 1, 1), 392 (M, 4), 349 (24), 307 (29), 293 (7), 243 (27), 228 (44), 202 (30), 149 (36), 135 (7), 118 (8), 107 (62), 91 (21), 77 (19), 65 (14), 43 (100), 39 (27).

*N-Acetyl-4-(p-tolylamino)-4-(2-(4-bromophenyl)thiazol-4-yl)butan-2-one* (**5h**): semisolid, yield: 80%, ^1^H-NMR (600 MHz, CDCl_3_) δ 7.67 (d, *J* = 8.4 Hz, 2H), 7.48 (d, *J* = 8.5 Hz, 2H), 7.21 (s, 1H), 7.11–6.73 (m, *J* = 139.3 Hz, 4H), 6.41 (t, *J* = 7.4 Hz, 1H), 3.05 (ddd, *J* = 24.6, 16.7, 7.5 Hz, 2H), 2.30 (s, 3H), 2.10 (s, 3H), 1.77 (s, 3H). ^13^C-NMR (151 MHz, CDCl_3_) δ 205.74, 170.90, 165.74, 155.98, 138.34, 137.28, 132.53, 132.00, 129.85, 129.45, 127.83, 124.10, 117.17, 51.35, 45.64, 30.17, 23.36, 21.08. GC-MS: *m*/*z* found: 456 (M calculated for C_22_H_21_BrN_2_O_2_S: 456); *m*/*z* (%) = 459 (M + 1, 5, ^81^Br), 458 (M, 6, ^81^Br), 457 (M + 1, 5, ^79^Br), 456 (M, 5, ^79^Br), 415 (25, ^81^Br), 413 (26, ^79^Br), 373 (26, ^81^Br), 371 (25, ^79^Br), 359 (9, ^81^Br), 357 (11, ^79^Br), 310 (6, ^81^Br), 308 (6, ^79^Br), 268 (13, ^81^Br), 266 (17, ^79^Br), 191 (10), 149 (45), 107 (48), 86 (68), 84 (99), 43 (100), 35 (34).

*N-Acetyl-4-(furan-2-yl)-4-(phenylamino)butan-2-one* (**5i**): yellow solid, mp: 80 °C, yield: 75%, ^1^H-NMR (600 MHz, CDCl_3_) δ 7.32–7.26 (m, 5H), 6.48 (t, *J* = 7.6 Hz, 1H) overlapped with 6.8–6.3 (m, 1H), 6.25–6.14 (m, 1H), 6.02 (s, 1H), 2.85 (ddd, *J* = 24.3, 16.0, 7.6 Hz, 1H), 2.15 (s, 3H), 1.73 (s, 3H). ^13^C-NMR (151 MHz, CDCl_3_) δ 205.31, 170.46, 152.24, 141.88, 139.27, 129.52, 129.30, 128.50, 110.40, 108.51, 48.48, 44.51, 29.85, 23.22. GC-MS: *m*/*z* found: 271 (M calculated for C_16_H_17_NO_3_: 271); *m*/*z* (%) = 271 (M, 1); 228 (3); 186 (3); 170 (3); 137 (2); 104 (1); 93 (6); 77 (8); 65 (12); 43 (100); 39 (32).

*N-Acetyl-4-(phenylamino)-4-(thiophen-2-yl)butan-2-one* (**5j**): white solid, mp: 84 °C, yield: 74%, ^1^H-NMR (600 MHz, CDCl_3_) δ 7.41–7.20 (m, 4H), 7.19 (d, *J* = 5.1 Hz, 1H), 7.06–6.65 (m, 1H), 6.85 (dd, *J* = 4.9, 3.6 Hz, 1H), 6.75 (d, *J* = 3.4 Hz, 1H), 6.56 (dd, *J* = 8.5, 6.6 Hz, 1H), 2.99 (ddd, *J* = 22.7, 16.3, 7.6 Hz, 2H), 2.17 (s, 3H), 1.74 (s, 3H). ^13^C-NMR (151 MHz, CDCl_3_) δ 205.36, 170.46, 142.75, 139.51, 129.78, 129.38, 128.63, 126.41, 126.23, 125.43, 50.29, 47.11, 30.05, 23.50. GC-MS: *m*/*z* found: 287 (M calculated for C_16_H_17_NO_2_S: 287); *m*/*z* (%) = 287 (M, 4); 244 (16); 202 (10); 188 (5); 153 (7); 135 (11); 118 (4); 109 (6); 93 (29); 84 (19); 77 (13); 66 (22); 43 (100); 39 (5); 27 (1).

*N-Acetyl-4-(phenylamino)-4-(pyridin-3-yl)butan-2-one* (**5k**): white solid, mp: 130 °C, yield: 75%, ^1^H-NMR (600 MHz, CDCl_3_) δ 8.71 (d, *J* = 2.0 Hz, 1H), 8.50 (dd, *J* = 4.7, 1.4 Hz, 1H), 7.72 (dd, *J* = 6.3, 1.6 Hz, 1H), 7.24 (dd, *J* = 7.8, 4.8 Hz, 1H), 7.12 (t, *J* = 7.9 Hz, 2H), 6.68 (t, *J* = 7.3 Hz, 1H), 6.53 (d, *J* = 7.8 Hz, 2H), 6.46 (t, *J* = 7.6 Hz, 1H), 2.92 (ddd, *J* = 22.4, 16.3, 7.6 Hz, 2H), 2.12 (s, 3H), 1.74 (s, 3H). ^13^C-NMR (151 MHz, CDCl_3_) δ 206.04, 170.2, 148.06, 139.82, 146.4, 137.8, 134.02, 128.93, 123.88, 118.17, 113.78, 52.08, 50.33, 30.93, 23.48. GC-MS: *m*/*z* found: 282 (M calculated for C_17_H_18_N_2_O_2_: 282); *m*/*z* (%) = 282 (M, 2); 239 (8); 183 (12); 149 (4); 132 (8); 106 (3); 93 (4); 77 (6); 43 (100); 39 (14); 27 (4).

## 4. Conclusions

We have successfully extended the lipase catalyzed direct Mannich reaction in the series of heterocyclic compounds. The presented results show that 2-arylthiazole-4-yl carbaldehydes, furane-2-carbaldehyde, thiophene-2-carbaldehyde and pyridine-3-carbaldehyde are novel substrates in the Mannich type condensation catalyzed by lipase B from *Candida antarctica*. By exploiting the biocatalytic promiscuity of CaL-B to catalyze the C-N and C-C bond formation, a series of eleven new heterocyclic Mannich bases and their N-acetyl derivatives were obtained in good yields (66%–80%), starting from the corresponding heterocyclic aldehydes, acetone and primary aromatic amines (aniline, *p*-toluidine). The newly obtained compounds were characterized by ^1^H-NMR, ^13^C-NMR and MS spectroscopy.
